# Transient blood–brain barrier disruption is induced by low pulsed electrical fields in vitro: an analysis of permeability and trans-endothelial electric resistivity

**DOI:** 10.1080/10717544.2019.1571123

**Published:** 2019-04-08

**Authors:** Shirley Sharabi, Yael Bresler, Orly Ravid, Chen Shemesh, Dana Atrakchi, Michal Schnaider-Beeri, Fabien Gosselet, Lucie Dehouck, David Last, David Guez, Dianne Daniels, Yael Mardor, Itzik Cooper

**Affiliations:** a The Advanced Technology Center, Sheba Medical Center, Ramat Gan, Israel;; b The Joseph Sagol Neuroscience Center, Sheba Medical Center, Ramat Gan, Israel;; c Sackler Faculty of Medicine, Tel-Aviv University, Tel Aviv, Israel;; d Department of Psychiatry, Icahn School of Medicine at Mount Sinai, New York, NY, USA;; e Blood-Brain Barrier Laboratory (LBHE), Université d’Artois, Lens, France;; f Interdisciplinary Center Herzliya, Herzliya, Israel

**Keywords:** Blood–brain barrier, pulsed electrical fields, electroporation, in vitro, trans-endothelial electric resistivity, permeability

## Abstract

The blood–brain barrier (BBB) is limiting transcellular and paracellular movement of molecules and cells, controls molecular traffic, and keeps out toxins. However, this protective function is the major hurdle for treating brain diseases such as brain tumors, Parkinson’s disease, Alzheimer’s disease, etc. It was previously demonstrated that high pulsed electrical fields (PEFs) can disrupt the BBB by inducing electroporation (EP) which increases the permeability of the transcellular route. Our goal was to study the effects of low PEFs, well below the threshold of EP on the integrity and function of the BBB. Ten low voltage pulses (5–100 V) were applied to a human *in vitro* BBB model. Changes in permeability to small molecules (NaF) were studied as well as changes in impedance spectrum and trans-endothelial electric resistivity. Viability and EP were evaluated by Presto-Blue and endogenous Lactate dehydrogenase release assays. The effect on tight junction and adherent junction protein was also studied. The results of low voltage experiments were compared to high voltage experiments (200–1400 V). A significant increase in permeability was found at voltages as low as 10 V despite EP only occurring from 100 V. The changes in permeability as a function of applied voltage were fitted to an inverse-exponential function, suggesting a plateau effect. Staining of VE-cadherin showed specific changes in protein expression. The results indicate that low PEFs can transiently disrupt the BBB by affecting the paracellular route, although the mechanism remains unclear.

## Introduction

The blood–brain barrier (BBB) is composed of brain endothelial cells, pericytes, and astrocytes and is located at the brain microvessel level. Interacting with other brain cells such as neurons and microglia, the BBB regulates molecules and cell exchanges between the periphery and the central nervous system (CNS) thus representing the major interface between brain and blood.

Endothelial cells of the BBB are different from those of extracranial tissues as they have continuous intercellular tight junctions (TJs) and adherens junctions (AJs) while they lack fenestrations. TJ associated proteins include occludin, claudin-5, and junctional adhesion molecules (Abbott et al., 2006; Abbott, 2013). Claudin-5 and Occludin are linked via zonula occludens (ZO) protein complexes to scaffolding proteins ZO-1, ZO-2, and ZO-3 that bind to the actin/myosin cytoskeletal system, resulting in modification of the TJs properties (Obermeier et al., 2013; van Tellingen et al., 2015). The endothelium acts as a dynamic barrier limiting transcellular and paracellular movement of molecules and cells and its main functions include control of molecular traffic and keeping out toxins (Abbott, 2013).

However, this protective function is the major hurdle for treating brain diseases such as brain tumors, Parkinson’s disease, Alzheimer’s disease, Huntington’s, etc. Currently, there is no state-of-the-art treatment approach for inducing BBB disruption despite extensive efforts. Mannitol injections into the internal carotid artery have been shown clinically to induce hemispheric BBB disruption, which is not localized and is accompanied by severe side effects including structural brain damage (Patel & Patel, 2017). Focused ultrasound (FUS) is the only noninvasive localized approach currently tested in initial clinical trials. Although showing promise, minor toxicity to the brain has been reported (Patel & Patel, 2017; Kovacs et al., 2018). On top of this, the optimal FUS parameters are determined individually during the treatment making it impossible to preplan the treatment (Downs et al., 2015), significantly affecting the treatment safety and outcome.

Another method for inducing transient, localized BBB disruption that was recently introduced is electroporation (EP). During EP, pulsed electrical fields (PEFs) are applied to cells or tissue, resulting in destabilization of the electrical potential across the cell membrane. The change in membrane potential results in creation of nanoscale aqueous pores in the lipid bi-layer, which in turn increases the permeability of the cell membrane. If the membrane re-seals again it is termed reversible EP and if the PEFs lead to cell death, it is termed irreversible EP (Weaver & Chizmadzhev, 1996). Our previous findings demonstrated the feasibility of obtaining EP-induced transient BBB disruption in rats scanned by contrast enhanced T1-weighted MRI following EP treatments (Hjouj et al., 2012; Sharabi et al., 2014). These results were corroborated in canines (Garcia et al., 2012) and the electric field threshold for EP-induced BBB disruption was found in these studies to be in range of 500–700V/cm. Bonakdar et al. and Lopez-Quintero et al. (Lopez-Quintero et al., 2010; Bonakdar et al., 2017) demonstrated *in vitro* increasing permeability of bovine aortic endothelial cells after application of 750,000 low voltage (1–10 V) pulses (90 µs pulses at 0.4 ms pulse interval for 5 min). They found increased hydraulic conductivity that was attributed to compromised continuity of the ZO-1 protein.

Here we applied only 10 low voltage pulses (ranging over 5–100 V) to a human *in vitro* BBB model (Cecchelli et al., 2014). We demonstrate that the BBB can be disrupted at such extremely low number of pulses and pulse amplitudes, well below the threshold for EP, thus evoking mechanisms involving changes in junctional proteins function rather than EP which induces poration of cell membranes.

## Methods

### Cells and BBB model

Human CD34+-derived endothelial cells and bovine brain pericytes from Artois BBB laboratory where their isolation and differentiation were conducted as previously described (Pedroso et al., 2011; Saint-Pol et al., 2012; Cecchelli et al., 2014). Regarding the collection of human umbilical cord blood: infants’ parents signed an informed consent form, in compliance with the French legislation. The protocol was approved by the French Ministry of Higher Education and Research (CODE-COH Number DC2011-1321). All experiments were carried out in accordance with the approved protocol.

**Figure 1.**
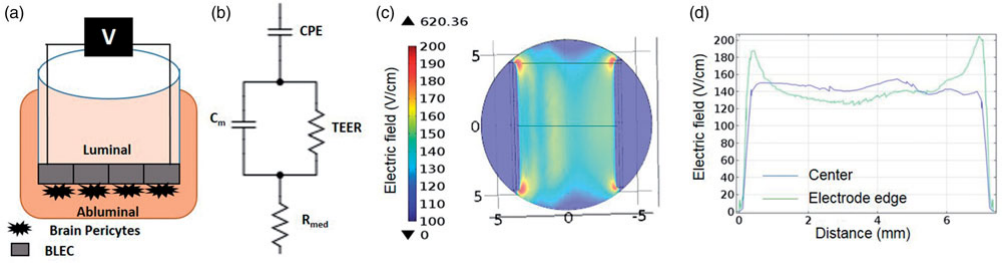
**(**A) Schematic presentation of the BBB model. (B) Scheme of a circuit consisting of the major contributors to impedance of the monolayer. (C) Geometry of numerical model and electric field distribution calculated using Equations 2–5 for 100 V. (D) Electric field distribution between the electrodes. Blue – center of electrodes, Green – electrodes edge. The electric field in the center is relatively uniform and can be approximated as voltage-to-distance ratio but around the edges higher electric fields are developed.

For each experiment, the cells were expanded on gelatin (Sigma, USA)-coated dishes in ECM medium. For co-culture experiments (BBB model), 5 × 10^4^ brain pericytes were seeded on the bottom of gelatin-coated Transwell (TW) inserts (3401-Costar, Corning, USA) and cultured in ECM medium. Human CD34+-derived endothelial cells were seeded at a density of 8 × 10^4^/insert onto the Matrigel-coated (BD Biosciences, San Jose, CA, USA) upper side of the 0.4 µm (1.12 cm^2^) TW. Cells were grown in co-culture for 6–8 days for permeability assays. During this time they acquired BBB properties and become brain-like endothelial cells (BLECs). A scheme of the model can be observed in Figure 1(A).

### Media

BLEC and pericytes were grown in ECM growth medium (Sciencell, USA) consisting of 5% fetal calf serum (Gibco, USA), ECGS supplements and 500 µg/ml gentamycin (Biological industries, Israel).

### PEFs protocol

PEFs were applied to TWs using a conventional electroporator power supply (BTX 830; Harvard Apparatus, Holliston, MA). Custom designed platinum iridium electrodes (0.68 cm apart, electrode length 0.9 cm) were used for all experiments. For each TW insert, 10 Pulses with a duration of 50µs pulses at 1 Hz were applied. Pulse amplitudes ranged between 5 and 100 V for low voltage experiments and 200–2000 V for high voltage experiments. For control, electrodes were placed inside the TW insert but no pulses were applied.

### Viability assay

In order to determine the viability of the cells, PrestoBlue^®^ reagent has been used as described in manufacturer’s protocol. Briefly, 35 µl Presto blue was added to the luminal side of the TW insert 1 h post PEFs application and the wells were incubated at 37 °C for 30 min. Media from Apical compartment was then read with Spectra Max fluorescence plate reader (560 nm excitation, 590 nm emission). *N* =  at least 6 TW inserts for each voltage amplitude conducted in 2–3 separate experiments.

### Endogenous lactate dehydrogenase (LDH) release assay for EP measurement

LDH assay was used to determine whether the PEFs induced EP in the BBB model. LDH is a stable cytosolic enzyme with a molecular weight of 144 kDa that is released from the cell upon membrane disruption. LDH kit (CytoTox 96^®^ Promega) was used for this assay. One hour post PEFs application, 50 µl of the apical medium of each TW insert was transferred to a 96 well plate and equal amount of CytoTox 96 Reagent was added to each well and incubated for 30 min. Stop Solution was then added, and the absorbance signal was measured at 490 nm with TECAN pro200 (Tecan Trading AG, Switzerland) plate reader. *N* =  at least three TW inserts for each voltage amplitude. The results were compared to the viability assay. If viability was not compromised, the PEFs protocol was considered to induce reversible EP. If the viability was affected, the treatment was considered to induce irreversible EP.

### Permeability assay

The permeability of the barrier to Sodium Fluorescein (NaF) was studied. Fifty microlitres of a solution containing 0.5 mg/ml NaF in PBS was added to the luminal side of each TW insert 1 min prior to PEFs application. Immediately after PEFs application, the plates were placed in a darkened incubator for 20 min with mild agitation. The fluorescence of the medium collected from the basal compartment was measured using TECAN pro200 plate reader (485/538 nm excitation/emission). In order to study the recovery of the barrier, the permeability assay was repeated 24 h later in the same inserts (the medium was replaced with fresh medium after the 20 min permeability assay). *N* = 6–12 TW inserts for each voltage amplitude.

The endothelial permeability coefficient (Pe) in cm/min was calculated as described in Vandenhaute et al. (Vandenhaute et al., 2012). In short, the clearance principle was used to obtain a concentration-independent transport parameter. The average volume cleared was plotted versus time, and the slope was estimated by linear regression. Both insert permeability (PS_f_, for insert only coated with Matrigel) and insert plus endothelial cell permeability (PS_t_, for insert with Matrigel and cells) were taken into consideration according to the following formula:

(1) $$\;\frac{1}{{\text{PS}}_{e}}=\frac{1}{\frac{1}{{\text{PS}}_{t}}-\frac{1}{{\text{PS}}_{f}}}$$

To obtain the endothelial permeability coefficient (Pe) of the molecules (in cm/min), the permeability value for the endothelial monolayer (Ps_e_) was divided by the surface area of the porous membrane of the insert.

### Impedance measurement

Impedance measurements offer the possibility to perform continuous real-time assessment of the effects of PEFs on the tightness of the BBB. Studying PEFs effects over a wide spectrum, rather than a single frequency, may provide additional information regarding the different processes that take place.

Impedance spectrum measurements ranging from 1 to 100 kHz were performed using a multi-well impedance spectrometer (cellZscope, Nano analytical, Germany). For each TW insert, the spectrum measurement lasted 36 s. Impedance was measured continuously for 24 h prior to the experiments. During the experiment, a TW insert, consisting of the luminal medium, was removed from the cellZscope into a 12 well plate containing growth medium and PEFs were applied. Exactly 1 min post PEFs application the TW insert was placed back into the cellZscope and impedance measure was continued for at least 10 min sequentially or until a plateau was reached. At this point another TW insert was removed from the cellZscope and so forth until all the TW inserts were treated. At the end of each experiment, continuous impedance measurement was performed for all TW inserts for additional 24 h while incubated at 37° and 5% CO_2_.

The modulus (|Z|) and the phase (θ) of the impedance were obtained for each frequency and equivalent circuit. The corresponding mathematical model was automatically applied by the cellZscope in order to extract the Trans endothelial electric resistivity (TEER) which is the resistivity of the barrier and is measured in Ω·cm^2^. The circuit can be seen in Figure 1(B). *C*_m_ is the membrane capacitance, TEER is the resistance across the transendothelial path, *R*_med_ is the medium resistance, and the CPE is a constant phase element representing the electrode capacitance. As long as EP of cell membrane does not occur, the conductance through the membrane is sufficiently small and the resistance of the membrane (*R*_m_) is neglected in this circuit. TEER is then extracted from the model and multiplied by the membrane area (1.12 cm^2^) and is presented in Ω·cm^2^. TEER values in the current study reached an average of 48 ± 4.4 Ω·cm^2^ before PEFs were applied.

### Staining for TJ and adherent junction proteins

Disruption of barrier function may be associated with changes in the expression and localization of TJ and AJ proteins. PEFs were applied to the cells with pulse voltages of 0 (control), 10, and 100 V. Cells were immunostained for the AJ protein VE-cadherin and the scaffolding proteins ZO-1, 30 min and 24 h post PEFs application. After PEFs application cells were fixed with ice-cold 4% paraformaldehyde for 10 min at 25 °C and exposed to a blocking solution (20% horse serum/0.1% Triton/phosphate-buffered saline) for 2 h. The cells were then incubated with goat anti VE-cadherin (Santa Cruz Biotechnology) and rabbit anti ZO-1 (Life Sciences) antibodies at a 1:200 dilution, overnight at 4 °C, washed with phosphate-buffered saline and stained with Cy3-labeled anti-rabbit or Alexa-Flour 488 anti-goat secondary antibodies (1:200, 1 h, room temperature). Nuclei were counterstained with Hoechst. The inserts were observed and photographed using a BX43 Olympus fluorescent microscope with a DP73 Olympus camera (Olympus America Inc., Center Valley, PA) at magnification of ×40 at similar exposure conditions. The images were converted to 8-bit images using image J software, and the thickness of the junctional proteins was calculated as the percentage of the stained area from the total field area. At least 10 pictures per/inserts were taken and 4–6 fields per picture were chosen for the analysis.

### Numerical model

Although we address the voltage applied on the active electrode throughout the manuscript, the observed physiological effects are actually induced by the electrical fields to which the samples are exposed to. This was done since the electric field is a more complex parameter that depends on the geometry of the experimental system and on the heterogeneity of the sample (Cemazar et al., 2018). In order to evaluate the induced electric field, a numerical model based on the geometry of the experiment was constructed (Figure 1(C)).

Finite element analysis software (COMSOL Multiphysics 4.3 b, Stockholm, Sweden) was used to calculate the electric field distributions within the *in vitro* BBB model. 3D geometry was constructed with dimensions equivalent to those in the experimental setup (Figure 1(C)). The TW insert was modeled as a cylinder of 6 mm radius and the electrodes were modeled as two parallel wires of 0.25 mm radius (0.68 cm apart, electrode length 0.9 cm). Electric conductivity of the electrodes was set to 5.278E + 06. The cells monolayer including the membrane of the TW insert was modeled as a 0.1 mm layer. The conductivity of the medium (1.04 S/m) was obtained by averaging the *R*_med_ (Ω) calculated by the CellZscope apparatus for the control experiments over 24 h and multiplying the result by *l*/*A*, where *l* is the height of the medium (0.44 cm) and *A* is the surface area of the TW insert (1.12 cm^2^).

The initial conductivity of the cell culture was calculated as the inverse of the average TEER (in Ω·m) prior to PEFs application and was set to 0.025 S/m.

The electric field was described by the Laplace equation for electric potential distribution in a volume conductor:

(2) ∇⋅(σ(E)∇φ)=0

where *σ* is the electric conductivity of the cell culture, *E* is the applied electric field and *ϕ* is the potential. Since the TEER is assumed to decrease due to the application of the electrical field, *σ(E)* dependence was included in the model. Since *σ* is the inverse of the TEER, the TEER experiments were used to determine the dependence by fitting the fold change in the TEER to a mathematical function and then multiplying the initial conductivity by the function 1/TEER(E).

Dirichlet boundary condition was applied to the surface of the electrode:

(3) $$\varphi =\varphi_{0}$$

and to the ground

(4) φ=0

where *ϕ_0_* is the applied potential on the active electrode.

The boundaries where the analyzed domain was not in contact with an electrode were treated as electrically isolative and Neumann boundary condition was set to zero on the outer border of the model:

(5) $$\frac{\partial \varphi }{\partial n}=0$$

where *n* denotes the normal to the boundary.

### Statistical analysis

Means are presented with the standard deviation, unless specified otherwise. Kolmogorov–Smirnov test was applied to the results to test the normality of the distribution. If the null hypothesis was rejected, a logarithmic transformation was performed on the data to achieve normal distribution. A one-way analysis of variance (ANOVA) was used to compare the effects of the different protocols. If Leven’s test of homogeneity was significant, Welch correction was applied and post hoc analysis was conducted by Games-Howell test. Otherwise post hoc analysis was conducted by Dunnett’ test and the results were compared to the control (0 V) group.

Curve fitting was performed using Matlab 11 A curve fitting tool. Goodness of fit was evaluated using *r*^2^ and Pearson correlation was used to study the correlation between TEER and permeability.

## Results

### Viability

**Figure 2.**
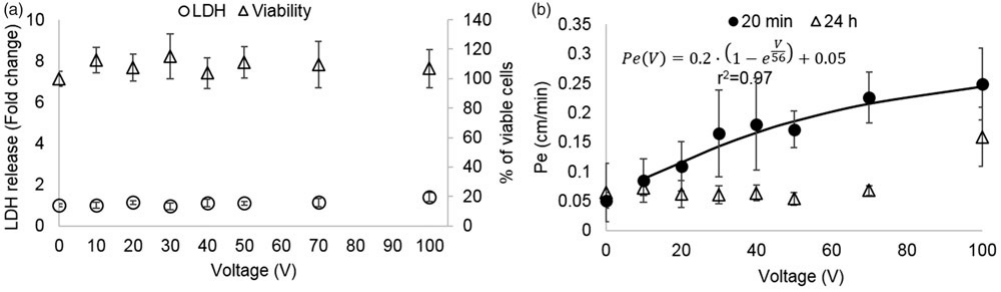
(A). Cell viability and EP as a function of the applied voltage. Viability is presented as % of viable cells and amount of EP indicated by LDH release normalized to control. It can be seen that there was no change in viability for all applied voltages. Increased LDH release occurring due to EP can be seen only at 100 V (*p < .0001). Data is shown as mean ± SD relative to control. (B) Pe coefficients for different treatment voltages (20 min and 24 h post PEFs application) and the fitted exponential curve for the 20 min permeability coefficients.

The viability of the cells treated with PEFs using pulse amplitudes of 1–100 V was assessed using the presto blue assay conducted 1 h post PEFs application. ANOVA indicated that there was no decrease in viability [*F*(7,56) = 1.6, *p* = .16] (Figure 2(A)), suggesting no irreversible damage.

### Electroporation

Since the viability assays revealed no cell death, we measured the LDH levels in the medium after PEFs application in order to assess whether BBB disruption can be explained by EP of the BLEC’s membranes. ANOVA with Dunnett *t*’ test post hoc analysis revealed a significant increase in LDH levels (by a factor of 1.4 ± 0.2) only in the 100 V group [ANOVA *F*(7,53) = 7.7, *p* < 2E-5, Dunnett *t*’ for 100 V group *p* < 5E-8] suggesting that BBB disruption could not be attributed to EP at PEFs lower than 100 V (Figure 2(A)).

### Low PEFs induce reversible permeability of the BBB

In order to assess the effects of PEFs on the integrity of the barrier the permeability coefficient Pe to the fluorescent molecule NaF was calculated for each treatment voltage. A 40 ± 9% increase in permeability was already visible at extremely low treatment voltages (10 V). The permeability continued to rise with the increase in treatment voltage. The results were fitted to an inverse exponent function (Figure 2(B), *r*^2^= 0.97, *p* < .0001), suggesting convergence towards higher voltages.

ANOVA was used to compare permeability coefficients Pe for each treatment voltage. Since Kolmogorov–Smirnov test rejected normality (*p* < .0001) a logarithmic transformation was performed, after which normality was obtained. Results of the ANOVA revealed a statistically significant main effect, *F*(7,97)=33, *p* < 5E − 24, *ω*^2^ =  0.73. Post hoc Dunnett *t*’ test indicated that there was a significant difference between the control group to each of the treatment voltages. The results are summarized in Table 1.

**Table 1.** Mean ± SD and *p* values of post hoc tests.

	0	5	10	15	20	30	40	50	70	100
Pe 30 min	0.05 ± 0.01	–	0.08 ± 0.03**	–	0.1 ± 0.04***	0.16 ± 0.07***	0.18 ± 0.07***	0.17 ± 0.03***	0.22 ± 0.04***	0.25 ± 0.06***
Pe 24 h	0.06 ± 0.04	–	0.07 ± 0.01 (0.86-1.2)	–	0.06 ± 0.02 (0.8-1.2)	0.06 ± 0.02 (0.86-1.3)	0.06 ± 0.01 (0.86-1.3)	0.05 ± 0.01 (0.89-1)	0.07 ± 0.007	0.15 ± 0.04*
TEER 30 min	1 ± 0.02	1 ± 0.03	0.85 ± 0.04**	0.75 ± 0.09***	0.62 ± 0.02***	0.47 ± 0.04***	–	0.43 ± 0.09***	–	0.28 ± 0.06***

*p < .05.

**p < .01.

***p < .0001.

The same analysis was conducted for the Pe coefficients calculated from the 24 h experiments. Since Kolmogorov–Smirnov test rejected normality (*p* < .0001) a logarithmic transformation was performed as described above, after which normality was obtained. Results of the ANOVA revealed a statistically significant main effect, *F*(6,60)=6.6, *p* < 1.7E − 20, *ω*^2^ =  0.81. These results show that approximately 81% of the total variation in permeability were attributable to differences in the treatment voltages. Post hoc Dunnett *t*’ test indicated that there was no difference between the control and the groups treated with voltages below 100 V, suggesting that the barrier function completely recovered at voltages up to 100 V but only partially recovered at 100 V. Results are summarized in Table 1.

### Low PEFs induce changes in impedance spectrum

**Figure 3.**
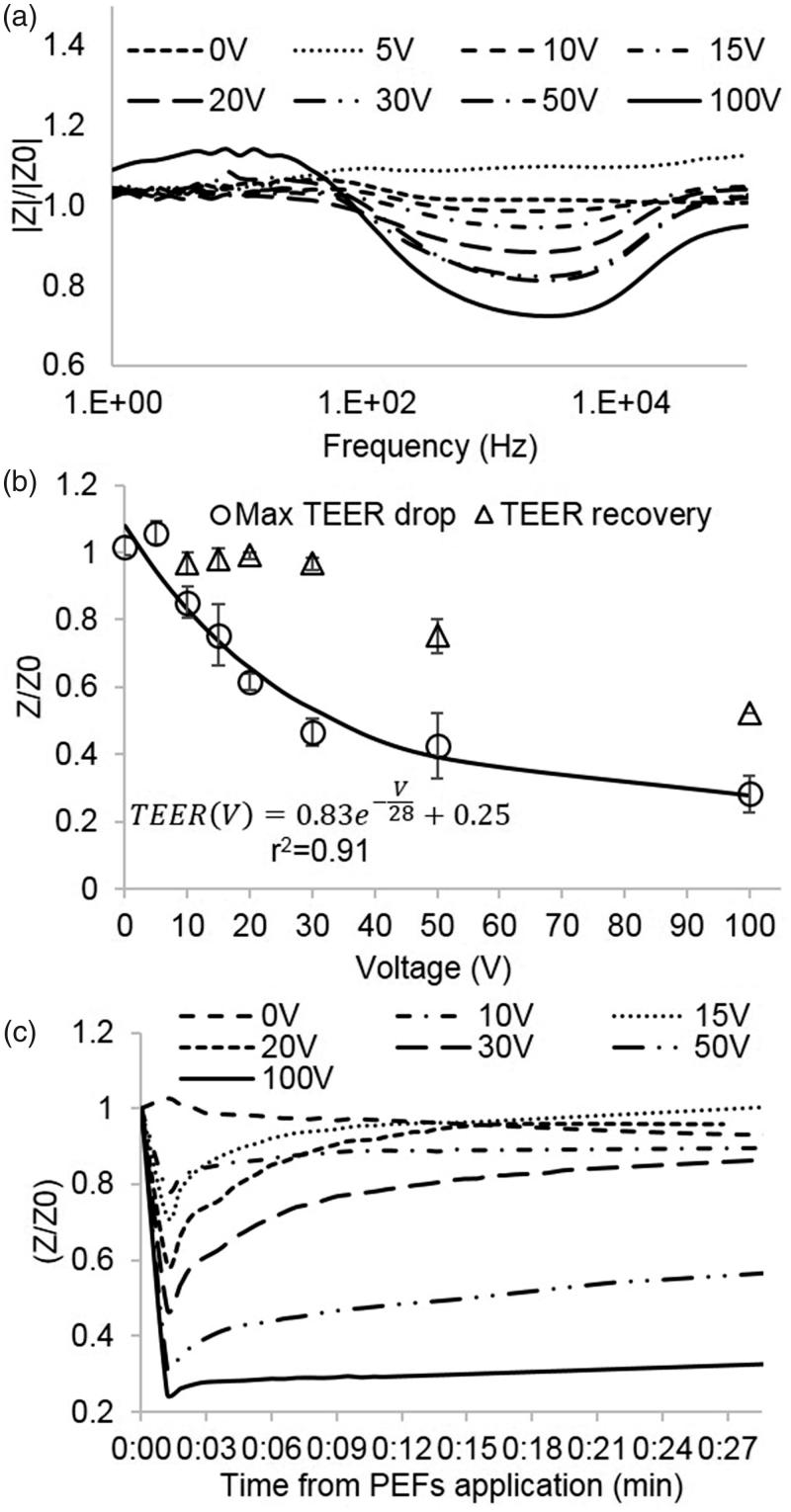
(A) Change in the modulus of the impedance immediately after PEFs application normalized to baseline as a function of the frequency for different PEFs amplitudes. The main effect is observed in the midrange frequencies. (B) Fold change in TEER immediately and 24 h post PEFs application as a function of the applied voltage. (C) TEER extracted from the impedance modulus and phase as calculated by the Cellzscope algorithm as a function of time post PEFs application.

The changes in barrier function were also evaluated by analysis of changes in impedance spectrum. No changes in the impedance spectrum were found in the control group or in the 5 V group. From 10 V and higher, shifts in the modulus and phase were observed for all the TW inserts (Figure 3(A)). A change in the modulus and phase was determined by comparing the amplitude and phase for each frequency immediately before and 1 min post PEFs application. Analysis of changes in the impedance spectrum revealed that PEFs does not influence the impedance at low frequencies (below 100 Hz) and that the maximal change in impedance occurred at mid-range frequencies between 1 and 5 KHz regardless of the applied voltage. This is in accordance with the concept of three distinct frequency regions in the impedance spectrum where the impedance is dominated by equivalent circuit elements: The low frequency range – dominated by the CPE, the mid frequency range – dominated by TEER, *R*_m_, and *C*_m_ and the high frequency range – dominated by *R*_med_ (Srinivasan et al., 2015).

### Low PEFs induce reversible decrease in TEER

TEER was extracted from the modulus and phase of the impedance spectrum measured 1 min post PEFs application. The results of the TEER experiments are consistent with the results of the permeability assay and clearly demonstrate a drop in TEER after PEFs application. The average TEER prior to PEFs application was 48 ± 4.4 Ω·cm^2^ while the average TEER after 100 V reduced to 8.6 ± 2.5 Ω·cm^2^

A significant decrease in TEER of 16 ± 5% was observed at treatment voltages as low as 10 V (no change at 5 V). The TEER continued to decrease with increasing treatment voltages. The results showed an exponential behavior (*r*^2^ = 0.95. *p* < .0001) suggesting convergence towards higher voltages. (Figure 3(B)).

ANOVA was used to compare fold change in TEER immediately post PEFs application for different treatment voltages. The test revealed a statistically significant main effect, *F*(7,18)=72.8 *p* < 6.6E − 12, *ω*^2^ =  0.94. These results show that approximately 94% of the total variation in TEER were attributable to differences in the treatment voltages. Post hoc comparisons, using the Dunnett *t*’ test demonstrated that TEER was significantly decreased compared to control in voltages starting from 10 V. The sizes of these effects are also described in Table 1.

The maximal TEER drop was observed at the first measured time point (1 min) after which the TEER gradually recovered. An example can be seen in Figure 3(C). A significant inverse correlation was found between the decrease in TEER after 1 min and the increase in permeability (Pearson, *r*^2^ = 0.91, *p* < .0001).

Three populations can be identified when observing the recovery: Full recovery (return to over 95% of baseline TEER) was observed in all TW treated with voltages below 50 V. Partial recovery (return to 75%±0.05% of baseline) of the TEER was obtained at 50 V and no recovery of TEER was observed at 100 V. The recovery process was completed within 30 min in most cases with no correlation between the time to recovery and the pulses voltage. Following the initial recovery, there was no significant change in TEER until the termination of the experiments 24 h post PEFs application.

### TJs and AJs alterations

**Figure 4.**
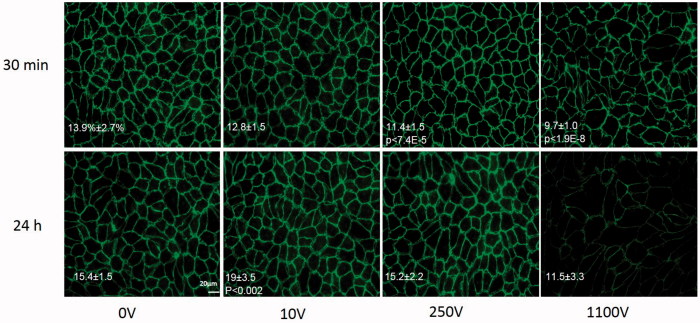
VE-cadherin immunostaining for different voltage amplitudes 30 min and 24 h post PEFs application. Images were captured with similar exposure conditions and magnification of ×400. Bar = 20 μm. Area coverage (percent from total area) 30 min and 24 h post PEFs application is presented on the images with significant p values compared to control.

The average percentage of area covered by VE-cadherin and ZO-1 in the control groups was found to be 13.9 ± 2.7% and 14.6 ± 1.7 respectively. ANOVA indicated that there was no change in the expression of both VE-cadherin and ZO-1 30 min post PEFs [*F*(2,30) = 1.3, *p* > .28 and *F*(2,12) = 0.65, *p* > .254, for VE-cadherin and ZO-1 respectively]. Surprisingly, at 24 h there was a significant increase in the percentage of VE-cadherin compared to control at 10 V [ANOVA with Welch correction *F*(2,26.06)=8.8 *p* < .001, Levens’ test F(1,42)=7.6, Games-Howell post hoc test *p* < .002] suggesting overexpression of the protein at 24 h. No change in expression was observed for ZO-1 [ANOVA with welch correction *F*(2,6.8) = 0.6, *p* > .57, levens’ test of homogeneity *F*(2,12) = 5.3, *p* > .02]. Despite the over-expression of VE-cadherin there was no significant change in permeability 24 h posttreatment compared to the control group (0.95 ± 0.12 of control, *t*-test, *p* < .7). Results of average coverage percent and significant *p* values are summarized in Table 1s in the supplementary file and in Figure 4.

### Numerical model

The numerical model calculated the electric field distribution between the electrodes. First the model was solved without conductivity changes. The results of the constant conductivity model demonstrated that in the center of the TW insert, the electric field between the electrode is relatively uniform and can be approximated as voltage-to-distance ratio. Around the edge of the electrodes, higher electrical fields are developed. In order to incorporate the changes in conductivity, the electric field of for each voltage was considered uniform and was calculated as the voltage-to-distance ratio. The time constant of the function describing the dependence of the change in TEER in the pulse voltage was modified accordingly (divided by 0.68 cm) and the equation was multiplied by the conductivity of the initial cells to account for the changes. The conductivity was thus described as:

(6) $$\sigma \left(E\right)=\frac{\sigma_{o}}{0.83e^{-\frac{E}{41}}+0.25}$$

where *σ*_0_ is the initial conductivity and *E* is the electric field.

The results of the model are presented in Figure 1(B,C).

### Comparison to high voltage PEFs

**Figure 5.**
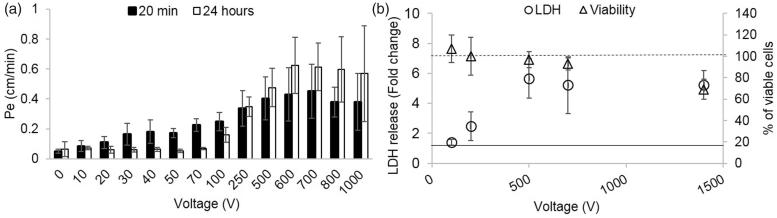
(A) Permeability coefficients at 20 min and 24h post PEFs application for 0–1000 V. At 100 V there is only partial recovery of barrier function and above 100 V deterioration of barrier function can be observed. (B) Fold increase in LDH levels (left axis, error bars are smaller than markers) and percentage of viable cells (right axis). Vertical lines represent the results of the controls (dashed line for viability and full line for LDH release).

In order to study the different effects of low and high PEFs on the integrity of the barrier, the permeability and TEER experiments were repeated using high PEFs ranging from 200 to 1400 V. The results are in accordance with the findings of the low voltage, meaning a plateau effect is observed at the high voltages. The results were fitted to the same functions that were used for 1–100 V which resulted in an increase in *r*^2^ for both the permeability (*r*^2^ = 0.95) and the TEER (*r*^2^ = 0.98). There was no recovery of either TEER or permeability for voltages above 100 V and a deterioration of barrier function can also be observed (Figure 5(A)).

ANOVA with Welch correction (since Leven’s test of homogeneity was significant (p<0.01) found no change in the viability of the cell below 1400 V using the Presto Blue assay. At 1400 V a decrease in viability of 30.8 ± 9% was observed. (ANOVA *F*(11,77) = 43, *p* < 1.9E−9 with Dunnet *t*’ post hoc analysis *p* < .8E−5 for 1400 V compared with control). Results of viability assays can be observed in Figure 5(B).

EP assessed by endogenous LDH release assay showed increased levels of LDH release at 100 V (1.4 ± 0.2). The levels continued to increase with the increase in the applied voltage reaching a maximum fold increase of ∼5 at 500 V and above (Figure 5(B)). Since viability assay revealed that cell death occurred at 1400 V, the results of LDH assay at 1400 V cannot be attributed solely to EP. In order to evaluate whether EP is the main effecter of barrier disruption when high voltages are applied, the correlation between the results of the LDH assay and permeability was examined for voltages ranging between 0 and 100 V and 100 and 700 V (1400 V was excluded due to cell death). The results indicate that there is a good correlation between the permeability and LDH release only when high voltages are applied, suggesting that from 100 V at least part of the increase in permeability can be attributed to EP (Pearson, 0–100 V *r*^2^ = 0.5, *p* < .003, 100–700 V *r*^2^ = 0.87 *p* < .0008)

The effect of high PEFs on VE-cadherin and ZO-1 expression levels was also studied. 30 min and 24 h post PEFs immunostaining for VE-cadherin was done after application of 250 V, 500 V, 800 V, 1100 V and 500 V for ZO-1. There was no difference between the expression levels of ZO-1 even at 500 V compared to control but a significant decrease in the expression of VE-cadherin was already observed at 250 V [ANOVA *F*(6,75) = 10 *p* < 3.7E − 8 with Dunnett post hoc analysis]. Results are summarized in Table 1s in the supplementary file and in Figure 4. An average decrease of 21 ± 5.6% in VE-Cadherin expression level was found and the decrease was not found to depend on the pulse voltages but rather there was no difference in expression levels between pulse voltages ranging between 250 and1100V. These results are also in accordance with the plateau effect observed in the high pulse voltages. At 24 h there is full recovery of expression levels at 250 V and 800 but not at 500 V and 1100 V [ANOVA with Welch correction *F*(2,42.8) = 9.6 *p* < 1E − 6, Levens’ test F(6,97) = 4.4, *p* < .001], post hoc Games-Howell test *p* values are summarized in Table 1s in the supplementary file (Figure 4).

## Discussion

The majority of currently available treatments for CNS diseases are ineffective due to low or no penetration of most therapeutic agents across the BBB (Patel & Patel, 2017). Thus, means to disrupt the BBB in a safe and controlled manner are in desperate need. There are two pathways for molecules to cross the BBB, the paracellular (between adjacent endothelial cells) and the transendothelial routes. It was previously demonstrated in vivo that PEFs can affect the transendothelial routes by means of EP (Garcia et al., 2012; Sharabi et al., 2014; Bonakdar et al., 2017). Thus, when high electric fields are applied, the cell membranes are electroporated and molecules can cross from blood vessels into the brain parenchyma. Our research hypothesis was that BBB disruption could occur at much lower field intensities, via the paracellular pathway rather than by EP.

Our objective was to study the effects of low PEFs (below the threshold of EP) on the function of an *in vitro* BBB model and to compare the effects with those of high PEFs. We applied 10 pulses at varying intensity from 5 to 1400 V to BLECs co-cultured with brain pericytes mimicking and modeling the human BBB, and studied the effects on the barrier function.

Our results clearly demonstrated increased paracellular barrier leakage even at pulses as low as 10 V, depicted both as decrease in TEER and as increase in permeability.

A correlation was found between the applied voltage and change in barrier function when measured by both TEER and permeability to small molecules such as NaF (376.3 Dalton). In both cases the effect was described by an exponential function with convergence towards high treatment voltages, suggesting a plateau effect.

### Mechanism of action – electroporation

In order to assess the mechanisms involved in PEFs induced BBB disruption, we first evaluated whether cell death or EP were involved. EP disrupts the BBB by inducing nanoscale pores in the lipid bilayer of the endothelial cells. EP may also increase the permeability of the monolayer and cause a decrease in the TEER values by inducing irreversible EP, meaning that the cell membranes will be disrupted in such a way that it will lead to cell death (Weaver & Chizmadzhev, 1996). The results of the viability study indicated that no cell death occurs as measured by the presto blue assay, but at the same time the levels of endogenous LDH secreted to the apical TW insert media was elevated (X1.4 from baseline) when 100 V pulses were applied to the cells (voltage-to-distance ratio of 147 V/cm). Since LDH assay is sensitive to membrane disruption, the elevation in LDH can be attributed to EP (Yao et al., 2002), meaning that only from 100 V the mechanism involved in BBB disruption can at least partially be attributed to EP. The levels of LDH secreted by the cells continued to increase with the increase in treatment voltages, suggesting an increase in the percentage of cells undergoing EP (Calderon-Miranda et al., 1999; Sharabi et al., 2016). The significant high correlation between the increased permeability and LDH levels that was found only above 100 V is strengthening the hypothesis that from 100 V, the increase in permeability is at least partly due to EP but below 100 V, other mechanisms are involved.

### Mechanism of action – effect on junctional proteins and cell scaffold

Changes in permeability can be induced by alterations in the expression levels or distribution of TJ and AJ proteins. Expression of VE-cadherin, which is a junctional protein very susceptible to various cellular stimuli and linked to hyperpermeability (Goddard & Iruela-Arispe, 2013) was not altered 30 min post low PEFs (0–100V) application. Interestingly, its expression at the cell–cell border has increased 24 h after PEFs application at 10 V, probably as part of the barrier cellular mechanisms to recover from the increased permeability occurred once PEFs were applied. Expression of ZO-1 was not altered 30 min or 24 h post PEFs application.

Attenuation in VE-cadherin 30 min post high PEFs application (above 250V) which induced EP, was found in our study when high voltages were applied. Kanthou et al. (Kanthou et al., 2006) and Markelc et al. (Markelc et al., 2018) also found a significant transient decrease of VE-cadherin immediately after application of EP. Markelc et al. (Markelc et al., 2018) applied 8 square wave pulses, 438 V – voltage-to-distance ratio of 600 V/cm, pulse length 100 μs at 1 Hz but contrary to our results found that the levels returned to baseline within 20 min of EP. Although they observed in some instances gaps between the endothelial cells after EP, which were still present even 20 min after EP. In our study, we found in all cases where EP occurred (above 250V) that the decrease in VE-cadherin lasted at least 30 min and that full recovery occurred after 24 h in some cases (250 and 800 V groups). The recovery after 24 h suggests reversibility of the EP process even though there was no recovery of either TEER or permeability at this time point. The recovery of the junctional proteins might indicate that permeability will recover eventually as well.

On the other hand, ZO-1 expression pattern was not altered, suggesting that PEFs-induced barrier alterations are protein specific. It is also possible that ZO-1 being a connector between extra membranous junctional proteins and the cytosolic cytoskeleton remains relatively stable as a cellular attempt of the endothelium to maintain barrier properties.

Another mechanism that was suggested by others to be involved in permeability changes of HUVECs is immediate dissolution of actin fibers and microtubules (Kanthou et al., 2006; Meulenberg et al., 2012) but these effects were never evaluated in pulses that are below EP threshold. Meulenberg et al. (Meulenberg et al., 2012) found after application of 8 square-wave electric pulses (1 Hz; duration 100 μs) that F-actin fibers and Beta-tubulin were unaffected below 273 V/cm and attributed the effects on the actin fibers and microtubules to EP.

It is possible that PEFs affect the function, rather than or in addition to the expression of the proteins. For instance, phosphorylation of VE-cadherin is associated with decreased junctional strength and increased BBB permeability (Dejana, 2004; Mishra & Singh, 2014). Although the effect of low PEFs on AJ and TJ proteins was not studied yet, the gap junction protein connexin was found to be phosphorylated after application of nano-second long high voltage pulses (Steuer et al., 2016).

The disruption may also be related to electrical forces, such as galvanotaxis (Li & Kolega, 2002; Uemura et al., 2016) or mechanical forces, for example, shear stress induced by the electric field, acting on the BLEC that are known to affect BBB permeability (Dejana, 2004; Nakadate et al., 2014). Thus, as of yet, the mechanism of action in which low PEFs disrupts the BBB remains to be elucidated.

### Mechanism of action – homogeneity of the effect and electric field distribution

The electric field distribution across the cells was calculated using the finite elements model. The results demonstrated that although in the center of the TW inset, the electric field is relatively uniform and can be described as voltage-to-distance ratio, the electric field distribution is not uniform across the monolayer, especially around the electrodes edges but still, at 100 V for example, only very small area adjacent to the electrodes tips is exposed to electric field above 200V/cm whereas most of the monolayer is exposed to electric field around 150 V/cm. Previous *in vivo* studies demonstrated EP-induced BBB disruption in electric field over 500V/cm (Garcia et al., 2012; Sharabi et al., 2016), One explanation for the difference can be that EP occurs at lower electric fields in the model. Second, an increase of relatively modest 1.4-fold in LDH release indicate that only part of the cells has been electroporated, possibly only the cells adjacent to the electrodes that are exposed to higher electric fields. Thus it is possible that several mechanisms including EP, junctional proteins alteration, microtubules changes etc. are involved in the increased permeability and above 100 V EP is more dominant than others.

### Plateau effect

The results of the high PEFs experiment support the finding of a plateau effect. There are a few probable reasons for this effect. The reasons can be attributed both to model limitation and to biological causes.

In the electrical model used to extract TEER from the impedance spectrum, the initial hypothesis is that *R*_m_ can be neglected as TEER≫*R*_m_. Nevertheless, this assumption is not valid if EP occurs and transcellular currents can also significantly alter TEER. As the TEER values mirror the integral resistance of the entire cell layer, if the paracellular resistance *R*_m_ has the same order of magnitude as the transcellular resistance *R*_t_, the current will distribute over the two current pathways. Thus, TEER needs to be replaced by a parallel combination of *R*_m_ and *R*_t_ (1/TEER =  1/*R*_t_+1/*R*_m_). The overall TEER will be smaller than either of its two components.

The results of the LDH experiments support the assumption that *R*_m_ cannot be neglected when pulses above 100 V are applied. Unfortunately, the two contributions cannot be determined separately by impedance spectroscopy alone (Wegener & Seebach, 2014). Nevertheless, this explanation does not explain the plateau observed in the permeability studies as well.

Another possible explanation for the convergence in the high treatment voltages also related to the induction of EP. EP induces transient cell swelling (Batista Napotnik & Miklavcic, 2018) restricting the paracellular pathway making the TEER predominated by the transcellular pathway. A corroboration for that can be found in the difference between time constants of the exponential functions describing the dependence of TEER and permeability in the applied voltage. Despite the excellent correlation, the time constants for the TEER equation is 28 and 56 for permeability, meaning the plateau for NaF permeability occurs at higher voltages.

Since the baseline TEER values were relatively low compared to the TEER of a human brain, and the TEER was reduced to close to zero even at relatively low voltages, it is possible that additional decrease was masked by this limitation of our BBB model.

Another possible explanation for the difference though, may be that TEER reflects the ionic conductance, whereas the flux of non-electrolyte tracers indicates the paracellular water flow, as well as the pore size between adjacent endothelial cells at the TJs (Deli et al., 2005).

### BBB *in vitro* model

Our study aimed for the understanding of the disruption of the BBB induced by low PEFs in order to develop a therapeutic tool to allow the delivery of drugs to the CNS. A BBB model based on human stem cell-derived endothelia cells co-cultured with brain pericytes was used. This reproducible and stable model is especially suitable for studying BBB disruption due to its high expression of TJs proteins and low permeability to small hydrophilic compounds (Cecchelli et al., 2014). Moreover, since the endothelial cells used in this model originate from human, this model facilitate the translation to clinical studies. A limitation of the model is the relatively low TEER values measured at baseline which was discussed above.

This is the first attempt to study the effect of PEFs on the BBB. Up until today, only a handful of studies have been conducted (Lopez-Quintero et al., 2010; Meulenberg et al., 2012; Bonakdar et al., 2017), focusing only on EP. On top of this, in most cases, the models used were not BBB models rather non-CNS blood vessels models which pose very distinct endothelium characteristics (Lopez-Quintero et al., 2010; Meulenberg et al., 2012).

### Clinical significance

Of clinical significance, full recovery of the barrier function was found below 50 V as can be observed in Figure 5. Both TEER and permeability recovered to baseline and the TEER experiments indicated fast recovery within 30 min from the treatment. Above 50 V partial recovery or no recovery of barrier function were observed. These findings support the hypothesis that the mechanism involved in PEFs induced BBB disruption depends on the treatment parameters. Nevertheless, it does not necessarily mean that no recovery will occur when PEFs will be applied *in vivo. In vivo* experiments conducted using MRI clearly demonstrated that BBB function recovers within 48 h (Hjouj et al., 2012; Sharabi et al., 2014). It is possible that not enough time elapsed from the PEF application or that barrier repairing molecules released from extra-cellular matrix (e.g. Laminin-10) (Kangwantas et al., 2016), adjacent astrocytes (Podjaski et al., 2015) or other NVU cells lead to recovery in the brain but are lacking in the *in vitro* system (Kangwantas et al., 2016).

## Conclusions

In conclusion, this is the first attempt to study the effects of low PEFs on the integrity of the BBB. The results of this study clearly demonstrate that it is possible to induce transient BBB disruption in vitro using PEFs by increasing the permeability of the paracellular-endothelial route. Yet there are still gaps in our understanding of the full mechanism of action that may involve several pathways depending on the treatment parameters. The results of this study may lay the foundation for new methods for increasing the efficacy of CNS drugs and for treating brain diseases such as brain tumors, and neurological disorders which will benefit from increased penetration of drugs into the brain. The reversibility of the disruption found in the low voltage is also an important aspect since long term disruption may lead to secondary problems.
